# Women’s satisfaction with childbirth care in Felege Hiwot Referral Hospital, Bahir Dar city, Northwest Ethiopia, 2014: cross sectional study

**DOI:** 10.1186/s13104-015-1494-0

**Published:** 2015-10-01

**Authors:** Mesafint Ewunetu Mekonnen, Worku Awoke Yalew, Zelalem Alamrew Anteneh

**Affiliations:** Department of Midwifery, College of Medicine and Health Sciences, Bahir Dar University, Po.Box 79, Bahir Dar City, Ethiopia; Department of Epidemiology, School of Public Health, College of Medicine and Health Sciences, Bahir Dar University, Po.Box 79, Bahir Dar City, Ethiopia

**Keywords:** Childbirth services, Women, Satisfaction

## Abstract

**Background:**

Globally, each year more than half million women die from pregnancy-related causes and an estimated 10 million experience injuries, infections and disease that can cause lifelong suffering attributed to poor quality care. Client satisfaction on health care delivery is an indication of quality care and reported to affect health service utilization. Individuals happy with the care received comply with services and follow up. The aim of this study was to assess women’s satisfaction with care during child birth and associated factors.

**Methods:**

Hospital based cross-sectional study was conducted on women delivering their baby in April to May 2014. Systematic sampling procedure was used to select 594 eligible women, and face to face interview technique was used to collect the data. The data were coded, entered into EPI-INFO (3.5.1) and analyzed using SPSS version 20 software package.

**Results:**

The findings of this study revealed that the proportion of women satisfied with childbirth care service was 74.9 %. Mothers whose age less than 20, and 20–34 years were less likely to satisfy with the care during child birth compared to mothers whose age was above 35 years (AOR = 0.17, 95 % CI 0.04–0.68, and AOR = 0.13.95 % CI 0.13–0.85). Besides, women who did not attend ANC were more than 3 times likely to satisfy with care compared to women attended ANC (AOR = 3.75, 95 % CI 1.12–12.59). Moreover, who who gave birth for the first time, and two to five times were more than 4 times likely to satisfy compared to women who gave birth for more than 5 times (AOR = 4.68, 95 % CI 1.75–12.54, and AOR = 4.38, 95 % CI 1.91–12.22).

**Conclusion:**

Only 75 % of women gave birth satisfied with the care they received. Moreover, age of women, antenatal care follow-up and the number of deliveries were important predictors of level of satisfaction. Therefore, the hospital administration and health professionals need to offer patient oriented service to increase level of satisfaction, as it is one of the measures of quality care.

## Background

Despite the effort and substantial amount of resources spent to reduce maternal mortality; every day, approximately 800 women die from preventable causes related to pregnancy and childbirth, and 99 % of all maternal deaths occur in developing countries [1, 2].

Each year more than half million women die from pregnancy-related causes, and an estimated 10 million experience injuries, infections, disease or disability. Moreover, a part of maternal mortality is attributed to poor quality of maternal care in health care institutions [3, 4].

Women in developing countries are disproportionately dying from pregnancy and pregnancy related causes; indicating that a woman’s lifetime risk of maternal death is 40 times higher in the developing countries compared to a woman in developed country [5]. Achieving the Millennium development goal for maternal health is impossible in Sub- Saharan Africa; Ethiopia is one of the six countries that contribute about 50 % of the maternal deaths worldwide [6].

It has been reported that several factors contributed to maternal deaths, one of which being a lack of skilled medical assistance and unattended delivery services that jeopardizes the lives of mothers and a newborn. Maternal satisfaction with hospital care during child birth is one of the many factors that play a role in the utilization of maternal health services [7, 8].

Studies declared that the magnitude of satisfaction on childbirth care varies across countries; a study conducted in Nairobi revealed that the overall satisfaction with childbirth care was 56 % [9]. A similar study conducted in central Ethiopia had shown that about 62.6 % of the women reported that they have been satisfied with their visit and the services utilization [10]. Another study conducted in Amhara region had revealed that the proportion of mothers satisfied with childbirth care service was 61.9 % [4].

It has been reported that satisfaction on health care delivery is an indication of quality care service [11–13]. Evidence had indicated that satisfaction affects health service utilization, individuals happy with the care received comply with services and follow up, and continue with the care [14, 15]. Despite women’s satisfaction with health care service is influenced by their expectations; a lot of factors affect women’s satisfaction during childbirth including interpersonal manner of care providers, technical quality of care, accessibility to care, birth outcomes, physical environment and availability of medical care resources [16]. Therefore, the aim of this study was to assess the magnitude and factors associated with women’s satisfaction during childbirth.

## Methods

A cross-sectional survey was conducted in Felege Hiwot Referral Hospital in Bahir Dar city. The estimated catchment population of the hospital is around 7 million people. The city is located in north western Ethiopia and the capital of the Amhara Administrative Region. Bahir Dar is situated at a distance of 565 km from Addis Ababa, the capital city of Ethiopia.

### Study population

The study populations were women who attended Felege Hiwot Referral Hospital to give birth between April and May 2014.

### Sample size determination

The sample size for this study was determined using single population proportion formula considering the assumptions: Proportion of women satisfied with childbirth care 61.9 % was taken from previous study [4], level of significance to be 5 % and margin of error to be 4 %. The final sample size with 10 % expected non response rate was 594 women gave birth.

### Sampling procedures

Based on 3 months report from the hospital preceding the survey, there were 3570 women received childbirth care in the hospital, which implies that on average 1190 women received childbirth care in each month, assuming that number of women attended the hospital for childbirth care; then the sample size 594 was divided to the total number of women received the care in each month to find the sampling fraction, and it was found one half (1/2). Then every other woman received childbirth care was recruited for the study.

### Measurement of level of satisfaction with childbirth care

We developed a list of ten indicators such as: availability of medical equipments, cleanliness and comfort of the waiting area, cleanliness of the facility, waiting time, access and cleanliness of toilet, respect and dignity, advice and information, encouragement and support during childbirth care, bed availability in the ward, and privacy were used to measure levels of satisfaction on childbirth care.

Responses on these indicators were recorded using a five scale liker scale (very dissatisfied, dissatisfied, neutral, satisfied, and very satisfied). Then, very satisfied and satisfied were merged as “satisfied” and neutral, dissatisfied and very dissatisfied were merged to “unsatisfied” for the sake of regression analysis [17, 18].

### Data collection tools and procedures

The data were collected using face to face administrated structured questionnaires adapted from reviewed literatures [4, 5, 7]. The questionnaire was initially prepared in English and was translated into Amharic local language in order to obtain the required information from the respondents.

Pre-test was done among 29 postnatal mothers at the Gondar university Hospital 1 month prior to the actual data collection period for the amendment of the questionnaires. Two data collector nurses were recruited and training was given for 2 days on the objectives of the study, method and the approaches they employee to collect data.

### Data processing and analysis

Prior to data entry, questionnaires were checked for errors, coded and were entered into EPI-INFO (3.5.1) and analyzed using SPSS version 20 software package.

During analysis, the responses of ‘very satisfied’ and ‘satisfied’ were classified as satisfied and responses of ‘very dissatisfied’, ‘dissatisfied’ and ‘neutral’ were classified unsatisfied. Neutral responses were classified as dissatisfied considering that they may represent a fearful way of expressing dissatisfaction. This is likely because the interview was undertaken within the hospitals and mothers may have been reluctant to express their dissatisfaction with the services they received.

The overall satisfaction level was calculated; and individuals scored 75 % and more of the items were categorized under “satisfied” and others as “unsatisfied”.

Univarate and bivarate analyses were computed to see the frequency distribution and to test whether there is an association between satisfaction and selected independent variables, respectively. Factors associated with satisfaction on bivariate were identified, and the variables with p value of 0.20 and less were taken to multivariable logistic regression analysis and the model was built with backward elimination.

Finally, 95 % confidence interval not including one with its corresponding p value less than 0.05 was considered statistically significant.

### Ethical consideration

Ethical clearance was obtained from the research and ethical review committee of GAMBY College of medical sciences. Permission letters were received from Amhara regional health bureau and Felege Hiwot Referral Hospital and verbal consent was obtained from each respondent before an interview.

## Results

### Socio-demographic characteristics of women received childbirth care in Felege Hiwot Hospital

A total of 594 women who received childbirth care were participated in the study. The mean and standard deviation of the age of women’s was 27 ± 5.3 years. Two hundred sixty-three (44.3 %) of mothers had no formal education. The majority (94.8 %) were married, 252 (42.4 %) were farmers. Three hundred seventeen (53.4 %) women came from urban areas. Five hundred and six (85.2 %) were Amhara by ethnicity. Five hundred and ten mothers were Orthodox Christian by religion. The mean household income of the respondents was 1317 ETB (see Table 1).

**Table 1:** Socio-demographic characteristics of delivering mothers’ in Felege Hiwot Referral Hospital, Bahir Dar city, Amhara Regional state, Ethiopia, April 1/2014–May 30/2014 total (n = 594)

Characteristics	Categories	N (%)
Age	<20	19 (3.2)
	20–34	500 (84.2)
	35–49	75 (12.6)
Ethnicity	Amhara	506 (85.2)
	Agew	82 (13.8)
	Other	6 (1.0)
Religion	Orthodox	510 (85.9)
	Muslim	60 (10.1)
	Protestant	24 (4.0)
Marital status	single	12 (2.0)
	Married	563 (94.8)
	Others	19 (3.2)
Educational status	No formal education	263 (44.3)
	Grade 1–6	50 (8.4)
	Grade 7–12	138 (23.2)
	Above 12	143 (24.1)
Occupation	Government employee	126 (21.2)
	Merchant	75 (12.6)
	House wife	128 (21.5)
	Farmer	252 (42.4)
	Others	13 (2.2)
Residence	Urban	317 (53.4)
	Rural	277 (46.6)

### The overall levels of women satisfaction with childbirth care in Felege Hiwot Hospital

**Fig. 1:**
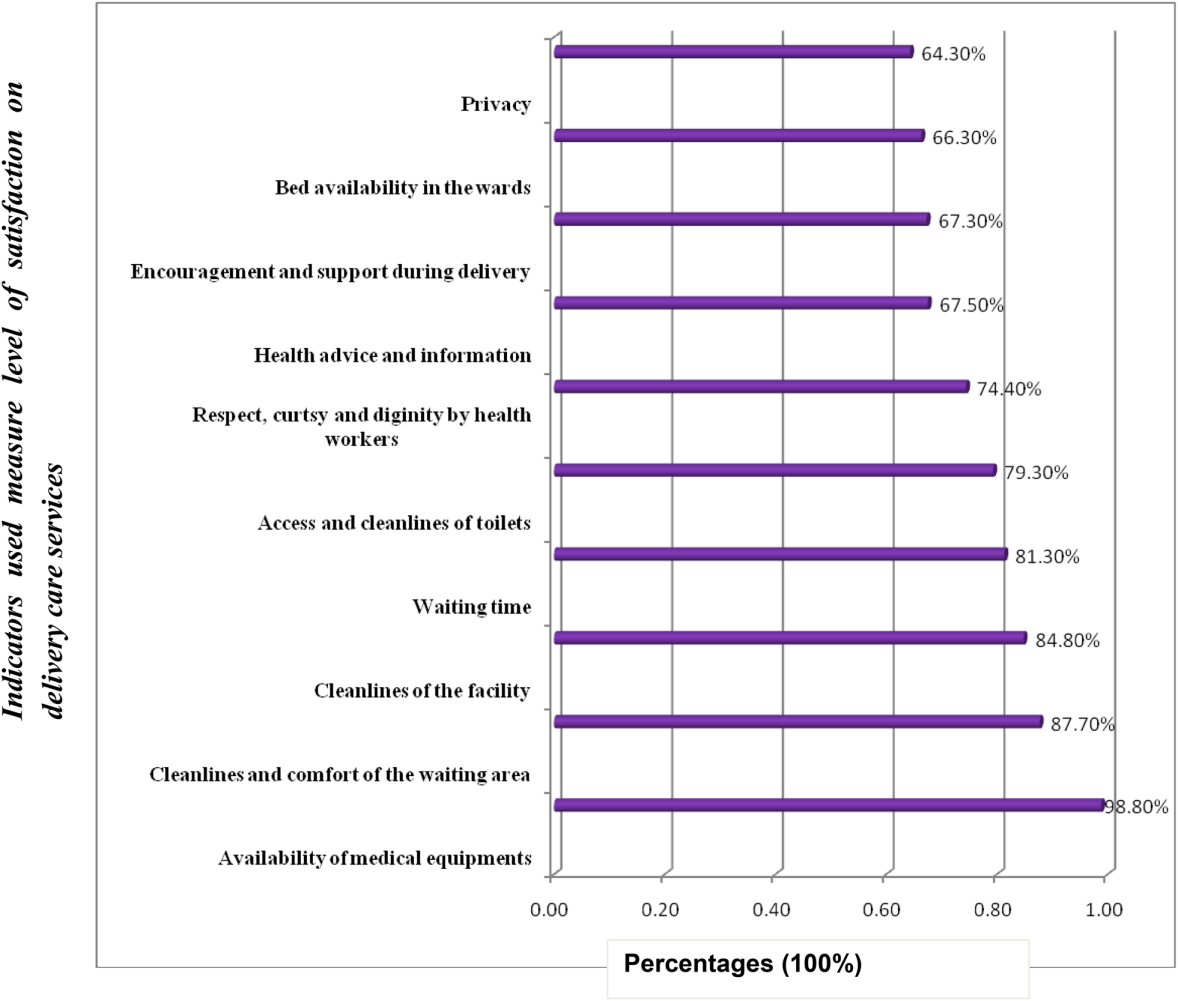
The percentages of items used to measure the overall satisfaction level of delivery care services for mothers delivered in Feleg Hiwot referral hospital, Bahir Dar city, April 1/2014–May 30/2014

The findings of this study revealed that the proportion of women satisfied with childbirth care was 74.9 %. Out of the indictors used to measure the overall level of satisfaction with childbirth care in this study, client privacy related satisfaction (64.3 %), availability of bed related satisfaction (66.3 %), encouragement and support during labor (67.3 %) and health advice and information given to the client related satisfaction (67.5 %) were the first least values (Fig. 1).

### The association between predictor variables and women’s satisfaction with childbirth care in Felege Hiwot Hospital, Bahir Dar city, April 1/2014–May 30/2014

In addition to the descriptive analysis, the bivariate and multivariable logistic regression analyses were computed. In the bivariate analysis age, educational status, occupation, income, attending ANC and number of gravidity were significantly associated with women’s satisfaction with childbirth care at p value of 0.2 levels.

In the multivariate logistic regression analysis age, ANC follow up, and the number of pregnancies were statically significant predictors of women’s satisfaction on childbirth care services at p value of <0.05.

Accordingly, women whose age less than 20, and 20 to 34 years were 0.174 and 0.131 times less likely to satisfy with the childbirth care services compared to women whose age from 35 to 49 years, respectively (AOR = 0.17, 95 % CI 0.04–0.68, and AOR = 0.13, 95 % CI 0.13–0.85).

Besides, women who did not attend ANC were more than 3 times more likely to be satisfied with childbirth care service compared to women those attended ANC follow up (AOR = 3.75, 95 % CI 1.12–12.59).

Moreover, women who gave birth for the first time were more than 4 times to satisfy with childbirth care compared to those gave birth for more than five times, and women gave birth for two to five times were about 5 times to satisfy from childbirth care they received compared to women gave birth for more than five times (AOR = 4.68, 95 % CI 1.75–12.54, and AOR = 4.83, 95 % CI 1.91–12.22) (see Table 2).

**Table 2:** Factors associated with mothers’ satisfaction with delivery services in Felege Hiwot Referral hospital, Bahir Dar city, April 1/2014–May 30/2014

Variables	Overall satisfaction		COR (95 % CI)	AOR (95 % CI)
	Satisfied	Dissatisfied		
Age				
<20	11	8	0.43 (0.15–1.25)	0.17 (0.04–0.68)*
20–34	375	125	0.95 (0.54–1.67)	0.33 (0.13–0.85)*
35–49	57	18	1.00	1.00
Educational status				
No formal education	189	74	0.766 (0.83–3.83)	1.13 (0.33–3.82)
Primary school	42	8	1.58 (0.70–1.77)	1.63 (0.49–5.37)
Secondary	104	34	0.92 (0.84–2.19)	0.86 (0.35–2.12)
College and above	110	33	1.00	1.00
Occupational status				
Gov’ employee	98	28	1.00	1.00
Merchant	56	19	0.84 (0.43–1.64)	0.93 (0.47–1.84)
House wife	102	26	1.12 (0.61–2.05)	0.92 (0.48–1.77)
Farmer	178	74	0.69 (0.42–1.13)	0.38(0.15–1.01)
Others	7	6	0.33 (0.33–7.51)	1.08(0.19–6.01)
Income				
<650	104	31	1.35 (0.76–2.25)	1.33 (0.75–2.34)
650–1000	125	51	0.99 (0.58–1.56)	1.06 (0.64–1.74)
1001–1900	112	25	1.81 (0.88–2.64)	1.91 (1.08–3.37)
>1900	104	42	1.00	1.00
ANC attending				
No	31	3	3.64 (1.10–12.10)	3.75 (1.12–12.59)*
Yes	414	146	1.00	1.00
No of gravidity				
One	144	50	1.08 (0.85–2.99)	4.68 (1.75–12.54)*
Two–five	344	78	1.65 (1.15–3.78)	4.83 (1.91–12.22)*
Above five	56	21	1.00	1.00

Asterisk shows the variable is significant at p-value of 0.05 level in the multivariable logistic regression analysis

## Discussion

The aim of this study was to determine the proportion of women satisfied with childbirth care and to identify the factors associated with satisfaction. Accordingly, this study revealed that the overall proportion of women satisfied with childbirth care was 74.9 %. This finding is supported by a study conducted in Assela Hospital among women who received delivery care, where the proportion of satisfaction was 80.7 % [19].

However, our finding is higher than studies conducted in Sri Lanka, Kenya, Amhara region on referral hospitals and Central Ethiopia, where the proportions of satisfaction on the childbirth care services were 48, 56, 61.9 and 62.6 % respectively [4, 9, 10, 16]. This variation may be due to difference in quality of services provided; an increase in awareness about what mothers should have obtained in the maternity care services.

The other probable reason for the difference might be exempted from any payment for childbirth care services, and an increase government concern for maternal health service in terms of qualified human power such as midwives and obstetricians. Besides, government focuses on comprehensive in-service trainings that might play for quality of service delivery [20]. The other probable reason why the level of satisfaction in this study higher than the others may be due to limitation of measuring levels of satisfaction, which means that level of satisfaction of women, could depends on many factors in addition to the indicators used to measure it in this study. The levels of satisfaction of women with childbirth care service depend on their expectation towards the service provision at the health institutions [21].

Regarding the analytic part of this study, age of the women was statistically significant predictors of women’s satisfaction childbirth care. Indicating that women whose age less than 20 and from 20 to 34 years were less likely to satisfy with the care they received compared to women whose age from 35 to 49 years (AOR = 0.17, 95 % CI 0.04–0.68, and AOR = 0.13; 95 % CI 0.13–0.85).

This finding is in accordance with other similar studies conducted in Zambia, Nigeria and Gondar (Ethiopia), where women of lower age group were less likely to satisfy with the health care service they received [22, 23]. The probable reason might be younger child bearing women didn’t have much experiences on the exiting hospitals’ service provision, this might played to expect much on the quality of the delivery care service.

The findings of this study also declared that attending ANC was the other significant predictor for women’s satisfaction with the childbirth care they received, women who didn’t attend ANC were more than 3 times more likely to satisfy compared to women who attended ANC (AOR = 3.75; 95 % CI 1.12–12.59). This is in line with similar findings in Sirilanka, Southern and central Ethiopia [10, 16, 24]. The probable reason might be due to the fact that the expectations of women childbirth care increases with their ANC follow up. This in turn demands enhanced health care services better quality childbirth care in the hospitals or health institutions.

Women who gave birth for the first time were more than 4 times likely to satisfy from the childbirth care they received compared to women who gave birth for more than five times (AOR = 4.68, 95 % CI 1.75–12.54).This is supported by other similar report where as a woman had greater numbers of pregnancies she is more likely to expect quality childbirth care service compared to a women gave birth for the first time [25]. This fact might be attributed to the experience of a woman on the childbirth care service increases the expectation and demand for more quality services. This is an indicator for the health institutions to improve quality of the childbirth care services in terms of different factors including qualified obstetricians and midwives.

## Conclusion

The findings of this study indicated that 75 % of women involved in the study were satisfied with childbirth care services in Felege Hiwot Referral Hospital. Whereas the remaining 25 % of the women unsatisfied with the care they received. Out of the indicators used to measure level of satisfaction, women’s satisfaction with privacy, availability of bed in the wards for delivery, encouragement and support during childbirth, and health advice and information provision were less than 70 %, which demands improvement.

Moreover, age of women, ANC follow up and the number of deliveries were important predictors of level of satisfaction. Therefore, the hospital has to work hard to increase levels of satisfaction as it is one of the measures of quality care delivery, specially improvement should be done to the privacy, availability of bed for childbirth, encouragement and support during childbirth, and health advice and information provision to increase the overall satisfaction and improve the quality of childbirth care. Besides, attention need to be given to ANC follow up and the quality of childbirth care that fulfills the women’s current demand.

## Abbreviations

ANC: antenatal care
AOR: adjusted odds ratio
ETB: Ethiopian Birr
CI: confidence interval
COR: crude odds ratio
MDGs: millennium development goals
SPSS: statistical package for the social sciences
